# Temporal fluctuations of tremor signals from inertial sensor: a preliminary study in differentiating Parkinson’s disease from essential tremor

**DOI:** 10.1186/s12938-015-0098-1

**Published:** 2015-11-04

**Authors:** Chusak Thanawattano, Ronachai Pongthornseri, Chanawat Anan, Songphon Dumnin, Roongroj Bhidayasiri

**Affiliations:** National Electronics and Computer Technology Center (NECTEC), National Science and Technology Development Agency (NSTDA), 112 Thailand Science Park, Phahonyothin Road, Khlong Nueng, Khlong Luang, Pathum Thani, 12120 Thailand; Department of Medicine, Faculty of Medicine, Chulalongkorn Center of Excellence for Parkinson Disease and Related Disorders, Chulalongkorn University and King Chulalongkorn Memorial Hospital, Thai Red Cross Society, Bangkok, 10330 Thailand

**Keywords:** Parkinson’s disease, Essential tremor, Tremor, Temporal fluctuation, Fluctuation ratio, Discrimination, Inertial sensor, Angular rate

## Abstract

**Background:**

Parkinson’s disease (PD) and essential tremor (ET) are the two most common movement disorders but the rate of misdiagnosis rate in these disorders is high due to similar characteristics of tremor. The purpose of the study is to present: (a) a solution to identify PD and ET patients by using the novel measurement of tremor signal variations while performing the resting task, (b) the improvement of the differentiation of PD from ET patients can be obtained by using the ratio of the novel measurement while performing two specific tasks.

**Methods:**

35 PD and 22 ET patients were asked to participate in the study. They were asked to wear a 6-axis inertial sensor on his/her index finger of the tremor dominant hand and perform three tasks including kinetic, postural and resting tasks. Each task required 10 s to complete. The angular rate signal measured during the performance of these tasks was band-pass filtered and transformed into a two-dimensional representation. The ratio of the ellipse area covering 95 % of this two-dimensional representation of different tasks was investigated and the two best tasks were selected for the purpose of differentiation.

**Results:**

The ellipse area of two-dimensional representation of the resting task of PD and ET subjects are statistically significantly different (*p* < 0.05). Furthermore, the fluctuation ratio, defined as a ratio of the ellipse area of two-dimensional representation of resting to kinetic tremor, of PD subjects were statistically significantly higher than ET subjects in all axes (*p* = 0.0014, 0.0011 and 0.0001 for x, y and z-axis, respectively). The validation shows that the proposed method provides 100 % sensitivity, specificity and accuracy of the discrimination in the 5 subjects in the validation group. While the method would have to be validated with a larger number of subjects, these preliminary results show the feasibility of the approach.

**Conclusions:**

This study provides the novel measurement of tremor variation in time domain termed ‘temporal fluctuation’. The temporal fluctuation of the resting task can be used to discriminate PD from ET subjects. The ratio of the temporal fluctuation of the resting task to the kinetic task improves the reliability of the discrimination. While the method is powerful, it is also simple so it could be applied on low resource platforms such as smart phones and watches which are commonly equipped with inertial sensors.

## Background

Parkinson’s disease (PD) is a progressive neurodegenerative disorder with the cardinal features of bradykinesia, rest tremor, rigidity, and postural instability [1]. There are reports stating that the diagnosis accuracy for Parkinson’s disease is only 76 % [2–4]. More recently, the accuracy of the clinical and pathological diagnosis of idiopathic Parkinson’s disease and another parkinsonian syndrome has been reported as 85.3 % (122 out of 143 cases) [5]. The common misdiagnosis includes parkinsonism-plus syndrome and essential tremor (ET) [6]. Although PD and ET appear to be two distinct diseases, clinical presentation of the two conditions can overlap, including tremor frequency range, the occurrence of resting tremors in ET (atypical ET syndrome) and the presence of postural tremors in certain tasks in PD [7]. The differential diagnosis of these tremors is important since the treatment depends on the specific etiology of each tremor type [8].

The diagnosis of PD is generally based on unified clinical diagnostic criteria in which the most widely accepted one is the United Kingdom Parkinson’s Disease Society Brain Bank (UKPDSBB). Based on this criteria, the diagnosis of PD can be made according to the presence of the cardinal features, including rest tremor, bradykinesia, rigidity, and postural instability, followed by the exclusion of other potential mimics. While tremor is an important clinical feature of PD, the diagnosis of PD cannot generally be made based on the features of tremor alone, but requires the presence of other cardinal features, with supporting clinical features if present.

Specifically on tremor in PD, they can have different manifestations although the most common type is the rest tremor, which accounts for 70 % of PD patients. However, in clinical practice, presentation of tremor in PD may overlap with other disorders, like essential tremor posing difficulties to clinicians to decide from the clinical examination alone if this tremor is parkinsonian. The common clinical scenario is to consider whether patients who have mild unilateral rest tremor, but also with postural tremor and unclear bradykinesia and rigidity if they are in fact parkinsonian. Since the evidence of PD treatment supports the fact that the treatment delays the disability [9], this clinical diagnosis of tremor alone can be challenging when presenting features of tremor overlap and characteristic features are not present, particularly in the early stage. Therefore, in our study, we seek to identify kinematic parameters that will enable us to differentiate tremor in PD from other disorders.

Current approaches of differential diagnosis of PD and ET can be categorized into conventional and non-conventional methods. The conventional approach includes clinical history and examination, supported by neuroimaging, and genetic studies if available, and further assessment of a response from antitremor medications. The non-conventional approach, which is more cost-effective and time efficient, is by studying tremor kinematics according to its intensity and frequency [8].

Recently, many researchers have been using, a particular or a combination of, low cost and widely available devices such as an electromyogram (EMG) [11, 12, 15, 16] and an accelerometer [10–19] to capture tremor signals from patients in order to discriminate PD, ET and physiologic tremors. Features of tremor signal are extracted by some general or specific methods such as peak frequency [10, 13], root mean square (RMS) of the linear acceleration [11, 12], approximate entropy [11, 12, 16], power spectral density [13, 14, 16], the shape of signal distribution [10, 12], wavelet coefficients [14, 16, 18] and higher order statistic parameters [14, 15]. These features, in general, are passed into one of the classifiers including neural networks [14, 18], support vector machines [17] and cluster analyses [10, 15], to classify the under test features as the group with the greatest potential. However, according to professional literatures, PD and ET cannot be confidently differentiated by previous methods.

In this report we proposed a novel method to extract a temporal feature based on the observation that, in PD patients, tremor frequencies have alteration while performing different tasks. This leads to the hypothesis that when PD patients perform a specific task with lower tremor frequency, they could potentially be observed to have more tremor fluctuation on that task. This feature, which is extracted from the angular rate tremor, can then be used to differentiate PD from ET subjects. Furthermore, the ratio of extracted features from an individual subject performing two specific tasks provides the improvement to the differential diagnosis.

## Methods

### Subjects

Fifty-seven subjects (35 PD, 22 ET patients) who were attending the outpatient clinic at the Chulalongkorn Center of Excellence for Parkinson’s Disease and Related Disorders, King Chulalongkorn Memorial Hospital, Bangkok Thailand, participated in the study. All PD and ET patients were instructed to withhold their medications for at least 12 h before the test. The study was approved by the Human Subjects Ethics Committee of the Faculty of Medicine, Chulalongkorn University. All subjects gave their written informed consent before entering the study in accordance with the declaration of Helsinki. Thirty-two PD and 20 ET patients were labeled as a training group whose data were used to generate the discrimination criteria. Three PD and 2 ET patients were labeled as a testing group whose data were used for validation purpose. Data from the two groups were collected separately where data of the training group was collected 2 years prior. There was no special criterion of patient selection for the training group. Demographic data and disease characteristics of participants in the training group are shown in Table 1. Patients with early stage or mild tremor, suggested by lower Fahn–Tolosa–Marin Tremor Rating Scale (TRS) compared to the training group, were selected as a testing group. Demographic data and disease characteristics of participants in the testing group are shown in Table 2.

**Table 1:** Demographic data of PD and ET subjects in training group

Demographic data	PD patients (mean ± SD)	ET patients (mean ± SD)	*p* value
Number (males)	32 (17)	20 (12)	0.64
Mean age (years)	65.50 ± 10.11	64.80 ± 15.76	0.85
Mean age at onset (years)	57.48 ± 10.71	55.30 ± 19.97	0.61
Disease duration (years)	7.60 ± 5.85	9.45 ± 9.63	0.39
TRS	37.31 ± 16.88	22.75 ± 12.10	0.0015
Hoehn and Yahr stage	2.42 ± 0.38		

*PD* Parkinson’s disease, *ET* essential tremor, *TRS* Fahn–Tolosa–Marin Tremor Rating Scale

**Table 2:** Demographic data of 3 PD and 2 ET subjects with mild tremor or early stage in testing group

Demographic data	PD 1	PD 2	PD 3	ET 1	ET 2
Gender	Male	Female	Male	Female	Male
Age (years)	76	61	60	81	84
Age at onset (years)	71	59	57	50	51
Disease duration (years)	5	2	3	31	23
TRS	21	19	13	14	17
Hoehn and Yahr stage	2	1.5	1.5	–	–

*PD* Parkinson’s disease, *ET* essential tremor, *TRS* Fahn–Tolosa–Marin Tremor Rating Scale

### Measurement

**Fig. 1:**
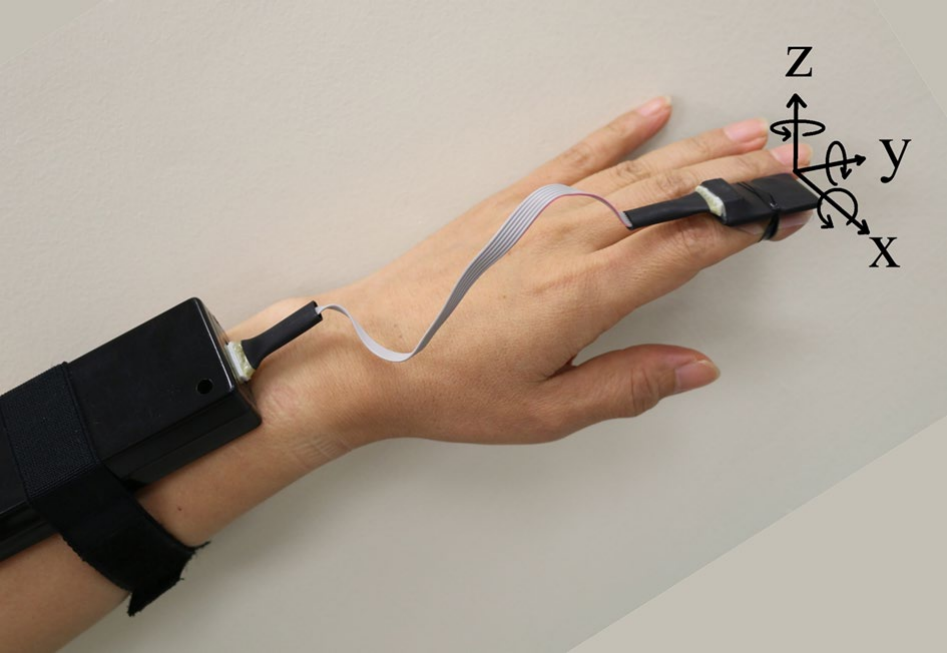
System set-up including a sensor unit and the transmitter, connected by a *thin* wire, and the axis orientation of an angular rate sensor

A 6-Degree of freedom (DOF) inertial measurement unit (IMU) with the capacity of measuring 125 samples/second of tri-axial acceleration and tri-axial angular velocity [20] is attached to subject’s upper limb during the experiment. However, only angular rate signals were considered in this study. The IMU is composed of a transmission part which is attached to subject’s wrist, and a sensor part which is attached to the index finger of the dominant hand manifesting tremor of the subject. The weight of the transmission part and the sensor part are 74 and 5 g, respectively. With this weight, the sensor part does not markedly affect the movement of a subject’s finger where the tremor is measured. The sensor orientation is shown in Fig. 1. For each subject, there are three tasks of data collection including kinetic, postural and resting tasks. For the kinetic task a participant was requested to do nose-target hand movement repeatedly. For the postural task, a participant outstretched his/her arms and hands forward from the shoulder. In the resting task, a participant was instructed to remain still with his/her arms on his/her laps with relaxed muscles. The signal was captured and transferred to a personal computer via wireless communication link. The in-house software was developed to collect inertial data of 10 s for each task and stored in a comma separated value (CSV) format for further analysis. The data can also be exported to other platforms.

### Signal preprocessing

**Fig. 2:**
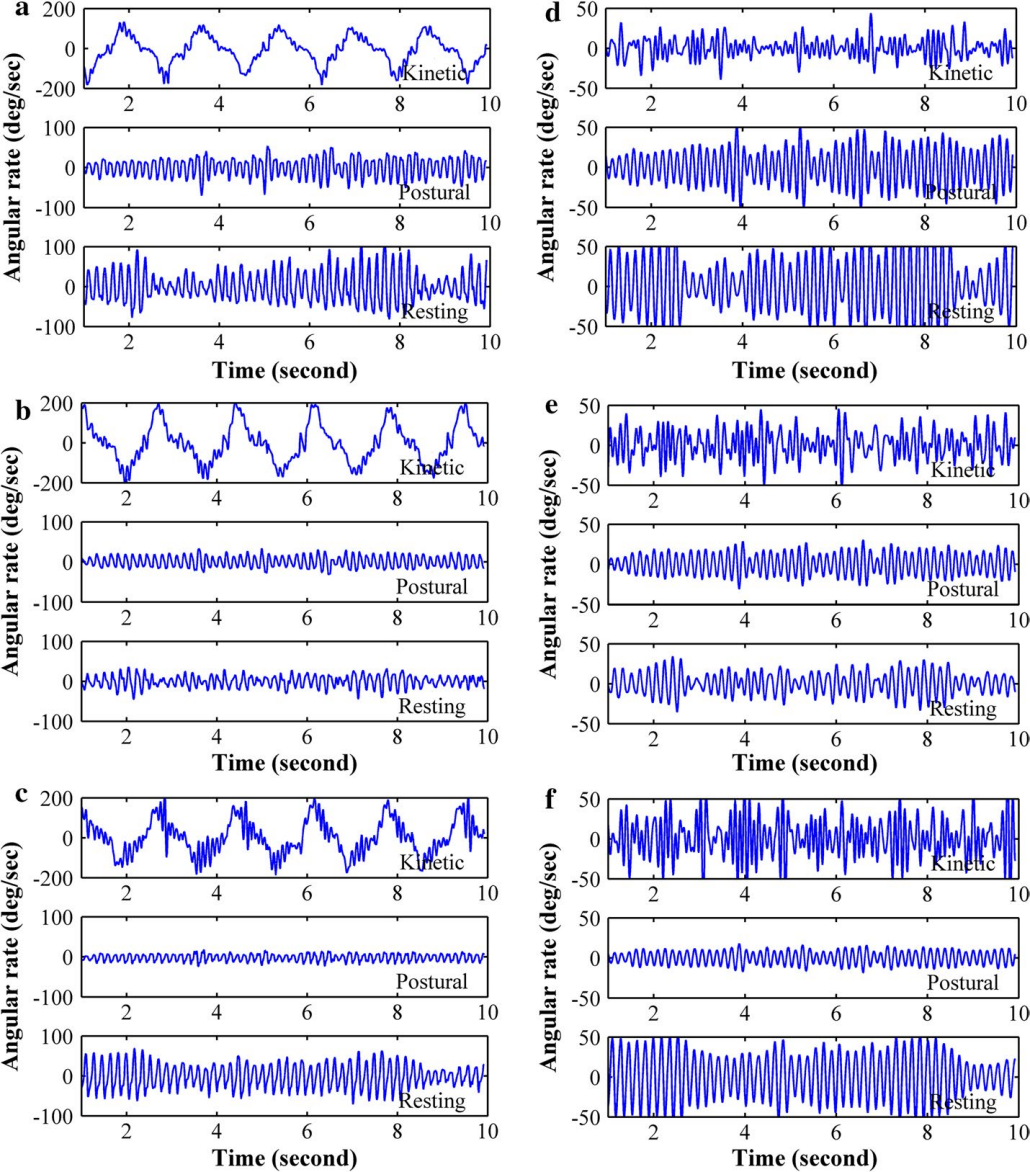
Tremor signal of a PD subject while performing kinetic, postural and resting tasks in x (**a**), y (**b**) and z-axis (**c**) and the corresponding preprocessed tremor signal (**d**–**f**). Subject was randomly selected

**Fig. 3:**
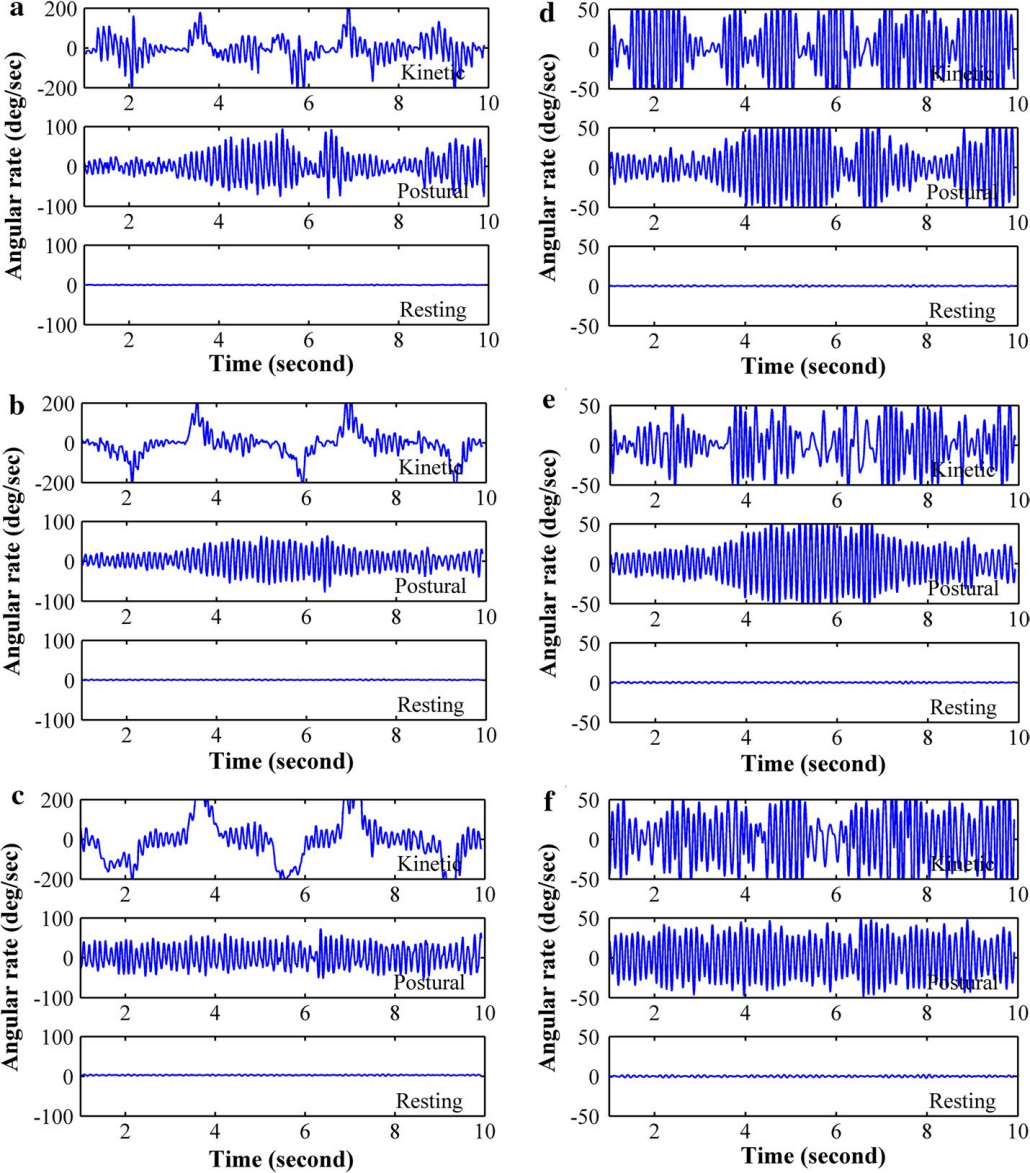
Tremor signal of an ET subject while performing kinetic, postural and resting tasks in x (**a**), y (**b**) and z-axis (**c**) and the corresponding preprocessed tremor signal (**d**), (**e**) and (**f**). Subject was randomly selected

The tremor signals obtained from patients were processed using Matlab (R2009b) running on a standard personal computer for preprocessing and analysis. Unless stated otherwise, a tremor signal is a time series obtained from the x-axis of an angular velocity sensor. Since tremor frequencies of PD and ET can overlap with range from 4 to 8 Hz [7], and to exclude low frequency high amplitude signal caused by movements while performing kinetic task, tremor signals were filtered with a 10th order Butterworth band-pass filter with cutoff frequencies of 3 and 10 Hz. Raw and preprocessed tremor signal collected from randomly selected PD and ET patients are shown in Figs. 2 and 3, respectively.

### Tremor characteristics

**Fig. 4:**
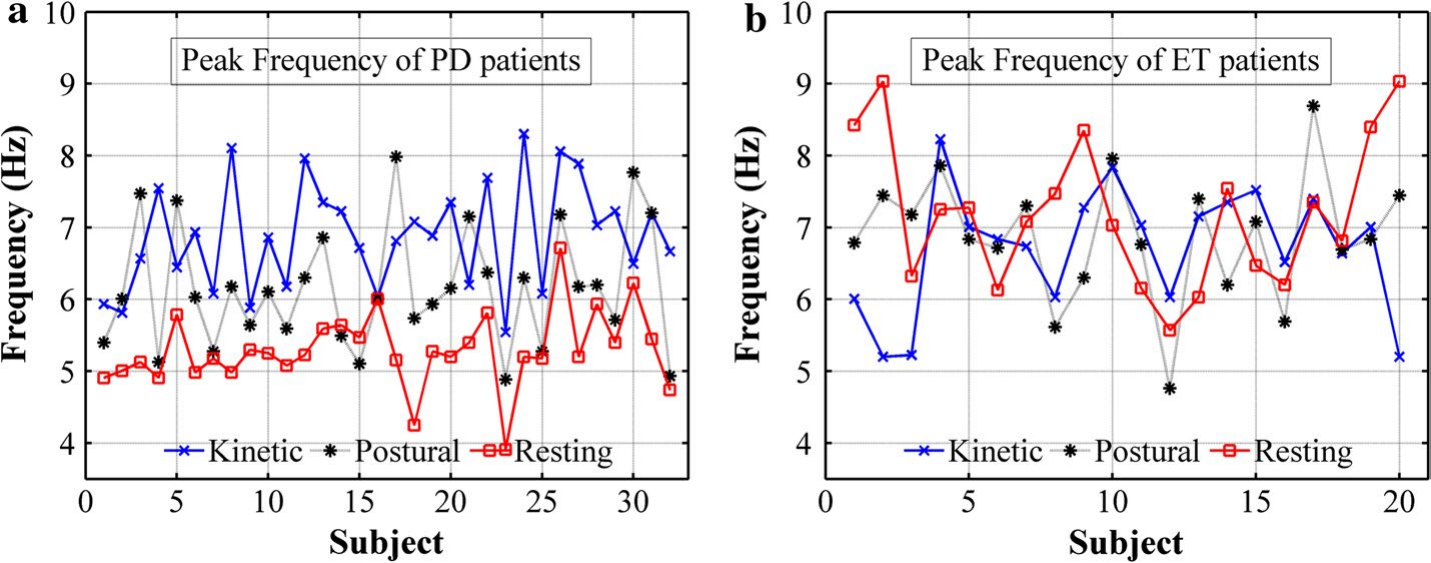
Average tremor peak frequencies of the training group, 32 PD (**a**) and 20 ET (**b**) subjects, while performing kinetic (*cross*), postural (*star*) and resting (*square*) task. Average peak frequency is the mean value of peak frequencies obtained from power spectral density of ten tremor subsequences. The power spectral density was obtained parametrically by autoregressive (AR) model parameters using Yule–Walker method

To investigate frequency characteristics of tremor signals while performing different tasks, filtered tremor signals were partitioned into ten equal-sized subsequences. Each subsequence is modeled with the 7th order [21] autoregressive (AR) process using Yule-Walker method. Model parameters of AR process were used to estimate the power spectrum of subsequences [22]. The peak frequency defined as the frequency achieved by the maximum of the power spectrum, was then located for each subsequence. Peak frequencies obtained from previous steps were averaged and used as a representation of the peak frequency of each 10-s long tremor signal. Average tremor peak frequencies of all PD and ET subjects while performing the three tasks can be shown in Fig. 4. Table 3 shows two-sample t test results of average peak frequencies between the two tasks.

**Table 3:** Two-sample t test results of peak frequencies of PD and ET patients in training group while performing different tasks

Tasks	PD patients	ET patients
Kinetic: postural	0.00054^a^	0.55
Kinetic: resting	9.92 × 10^−14a^	0.11
Postural: resting	9.78 × 10^−6a^	0.30

^a^Denotes statistical significance

### Temporal fluctuation

Even though frequency alteration from kinetic to resting tasks of PD subjects tend to be on the lower side as shown in Fig. 4, this does not mean that this feature can solely be used for perfectly discriminating PD from ET because it is only sensitive to PD but not to ET as two-sample t test results shown in Table 3. To discriminate PD and ET, one needs to consider both frequency and time domains. To acquire features that are composed of both domains, previous literatures [14, 16, 18] applied a transformation such as the wavelet transform which decomposes time series signal into time and scale in different resolutions. Some research groups separately acquire frequency and temporal features by using peak frequency [10, 13], RMS [11, 12], approximate entropy [11, 12, 16], power spectral density [13, 14, 16] and higher order statistical parameters [14, 15] as features. These features are then fed to a classifier such as a neural network or a support vector machine. However none of aforementioned methods provides perfect discrimination.

To solve this problem, we generated a new relation according to signal amplitude temporal variation as follows. Suppose *s(n)* is a signal acquired from inertial sensor at *n*th sample and *d*_*1*_ and *d*_*2*_ are delay units which *d*_*1*_ < *d*_*2*_. Let $f\left( n \right) = (x\left( n \right),y(n))$ be a two-dimensional signal representing a relation of tremor samples at different delay units *d*_*1*_ and *d*_*2*_ as

1 $$f\left( n \right) \, = \, \left( {x\left( n \right),y\left( n \right)} \right) \, = \, \left( {\left( {s\left( {n + \, d_{1} } \right) - s\left( n \right)} \right), \, \left( {s\left( {n + \, d_{2} } \right) - s\left( n \right)} \right)} \right).$$

**Fig. 5:**
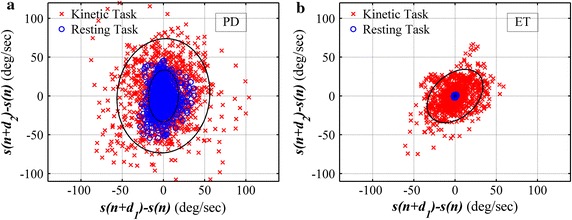
Two-dimensional plot of relation (1) of tremor signal obtained while performing kinetic (cross) and resting (*circle*) tasks from a PD (**a**) and an ET (**b**) patient. Both are males with 77 years of ages and 7 years of disease duration. Delay units *d*_*1*_ and *d*_*2*_ in relation (1) were involuntarily selected to 5 and 20, respectively. The temporal fluctuation of tremor is the area of 95 % confidence *ellipse* covering the two-dimensional representation of kinetic (*filled line*) and resting (*dash line*) task

Since filtered tremor frequencies range from 3 to 10 Hz, tremor signals could have maximum fluctuations at the delay unit setting from approximately 0.05–0.16 s. For example, *d*_*1*_ and *d*_*2*_ can be selected to 5 and 20 samples, respectively. Therefore, we can generate a two-dimensional plot of the relation (1) with of tremor signals obtained while performing kinetic and resting tasks from a PD and an ET patient as shown in Fig. 5. The ‘temporal fluctuation’ of tremor (*TF*) can be quantitatively defined as the area of 95 % confidence ellipse covering *f(n)* as shown in Fig. 5.

### Fluctuation ratio

It is common for a PD subject to be visually observed to have rest tremor. The tremor amplitude is reduced when a subject has motor action such as moving or stretching his/her arm in most cases. This phenomenon is the opposite for an ET subject. Therefore should there be a feature that compares the aforementioned characteristic of tremor during resting and kinetic tasks, the ratio of these measurements could provide insights to increase the reliability of the discrimination. As shown in Fig. 4, the largest variation of average peak frequencies is found when a PD subject performed a kinetic task and then a resting task. The differentiation of PD from ET can then be improved by using features related to the alteration of tremor peak frequencies of these two tasks. Concerning an individual subject, the lower tremor frequency corresponds to the higher tremor amplitude, and vice versa. Therefore, the hypothesis is that PD subjects have more temporal fluctuations in a resting task than in a kinetic task.

To differentiate PD and ET, based on tasks performed by an individual, we defined the ‘fluctuation ratio’ (*RF*) as a proportion of temporal fluctuations of a resting task to a kinetic task of a particular subject as in (2).

2 $$RF \, = \, log\left( {\left( {100 \times TF_{resting} } \right)/TF_{kinetic} } \right)$$

The higher the fluctuation ratio the greater is the potential that the tremor belongs to PD subjects. By this quantitative definition, we can then define a threshold value to discriminate between PD and ET subjects.

## Results and discussion

**Fig. 6:**
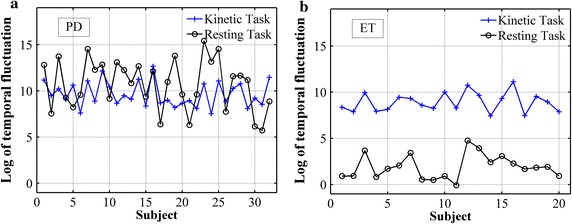
The temporal fluctuation of the training group, 32 PD (**a**) and 20 ET (**b**) subjects, while performing kinetic (*plus*) and resting (*circle*) tasks. Delay units *d*_*1*_ and *d*_*2*_ in relation (1) were involuntarily selected to 5 and 20, respectively. The temporal fluctuation is shown in *log scale*

**Fig. 7:**
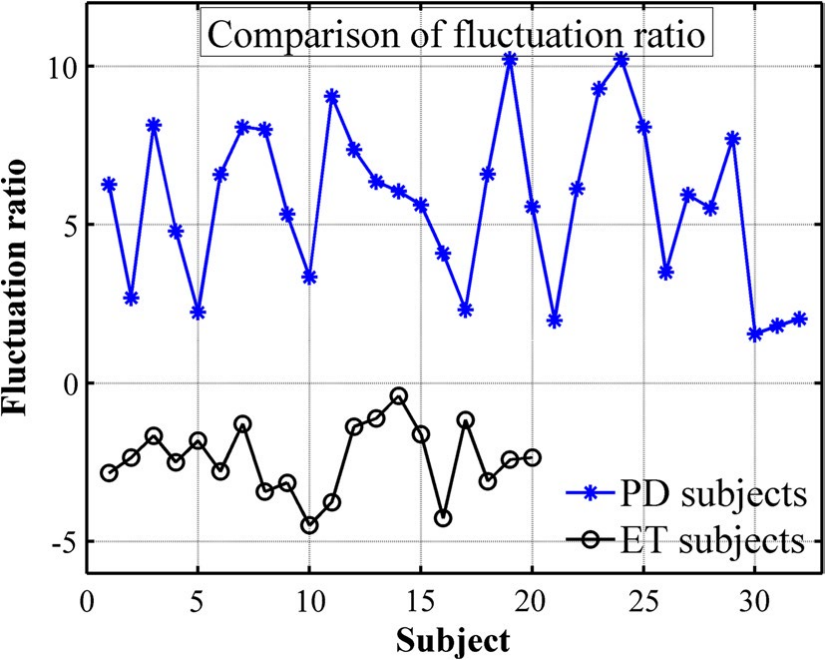
The fluctuation ratio with delay unit *d*_*1*_ = 5 samples and *d*_*2*_ = 20 samples in relation (1) of the training group, 32 PD (*star*) and 20 ET (*circle*) subjects. The fluctuation ratio is a log scale of percentage of temporal fluctuation while performing resting task to kinetic task of an individual subject. The log scale is used because the distribution of percentage of temporal fluctuation while performing resting task to kinetic task is skew

**Fig. 8:**
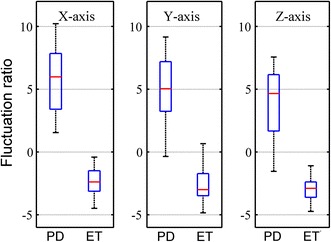
The distribution of fluctuation ratios of the training group, 32 PD and 20 ET subjects, obtained from x, y and z axis of angular rate sensor

To investigate the common outcome, delay units *d*_*1*_ and *d*_*2*_ are involuntarily selected to 5 and 20, respectively. The temporal fluctuation of PD and ET subjects are shown in Fig. 6. Matlab performed two-sample t test to compare the averages between two groups and determine if there is a significant difference between them. It can be seen that PD and ET subjects can be differentiated based on the temporal fluctuation measured while performing the resting task (*p* = 0.0492). Furthermore, it can be visually observed that the temporal fluctuation of ET subjects while performing resting and kinetic task are more statistically significantly different (*p* = 0.0013) than PD subjects (*p* = 0.0228). The fluctuation ratio with selected delay units of PD and ET subjects are shown in Fig. 7 (*p* < 0.01). To compare fluctuation ratios obtained from different axes, the distribution of fluctuation ratios in all axes is shown in Fig. 8. It can be seen that only fluctuation ratios in the x-axis have a statistically significant separation between the lowest fluctuation ratio from PD subject and the largest fluctuation ratio from ET subjects. This is because a subject with tremor has more angular movement around the x-axis than other axes. The list of delay units tested in the experiment and their two-sample t test results and separation distances between fluctuation ratio of PD and ET groups are shown in Tables 4 and 5, respectively. However, two-sample t test results of the x-axis have larger values comparing to the other two axes because the fluctuation ratios of the x-axis of PD group vary in a larger scale. The largest statistical difference of PD and ET (*p* = 0.0014) is obtained when *d*_*1*_ and *d*_*2*_ are selected to 15 and 40, respectively. The largest separation distance between fluctuation ratio of PD and ET groups is obtained when *d*_*1*_ and *d*_*2*_ are selected to 5 and 20, respectively. Regarding the tremor signals collected from the training group, the fluctuation ratio obtained from an ET subject is always negative (equivalent to 1 % in normal scale) and the fluctuation ratio obtained from a PD subject is always positive. Therefore, the threshold ratio could be simply set to zero and the classification rule can be assigned as follows. A patient is classified as a PD subject when his/her fluctuation ratio is greater than zero and a patient is classified as an ET subject when his/her fluctuation ratio is less than zero.

**Table 4:** Two-sample t test results of fluctuation ratios between PD and ET patients in training group in three axes with a set of different values of delay units *d*_*1*_ and *d*_*2*_

*d*_*1*_ (samples)	*d*_*2*_ (samples)	X-axis	Y-axis	Z-axis
5	10	0.0111	0.0035	0.0001
5	20	0.0097	0.0021	0.0003
10	20	0.0111	0.0022	0.0002
10	30	0.0107	0.0021	0.0003
15	30	0.0317	0.0072	0.0002
15	40	0.0014	0.0011	0.0003
20	30	0.0151	0.0033	0.0003
20	40	0.0057	0.0015	0.0004

**Table 5:** Separation distances of fluctuation ratios between PD and ET patients in training group in three axes with a set of different values of delay units *d*_*1*_ and *d*_*2*_

*d*_*1*_ (samples)	*d*_*2*_ (samples)	X-axis	Y-axis	Z-axis
5	10	1.0747	−0.5290	−1.0970
5	20	1.9436	−1.0208	−0.4472
10	20	1.9435	−0.9816	−0.9146
10	30	1.1455	−1.2763	−1.1133
15	30	0.8109	−1.3183	−0.5249
15	40	1.5539	−0.7817	−1.2615
20	30	1.2872	−1.3904	−0.9399
20	40	0.8653	−1.9096	−0.8077

To validate the discrimination performance, the signals collected from the testing group (3 PD and 2 ET) were processed as described procedure. Fluctuation ratios of PD patients in testing group are 0.7298, 0.0679 and 0.2788. Fluctuation ratios of ET patients in testing group are −3.1430 and −1.8253.

Suppose PD is a positive case and ET is a negative case, the performance of the discrimination is evaluated by sensitivity, specificity, and accuracy, as follows:

3 $$Sensitivity\,\,\left( \% \right) = \frac{TP}{TP + FN} \times 100$$

4 $$Specificity\,\,\left( \% \right) = \frac{TN}{TN + FP} \times 100$$

5 $$Accuracy\,\,\left( \% \right) = \frac{TN}{TP + TN + FP + FN} \times 100$$

where *TP* is true positive, *TN* is true negative, *FP* is false positive and *FN* is false negative. Despite a small number of patients in testing group, they were considered having mild tremor or early stage suggested by low TRS. The discrimination of PD from ET by this method provides 100 % accuracy, sensitivity and specificity in the 5 subjects in the validation group.

## Conclusions

Parkinson’s disease and essential tremor are among the most frequent movement disorders. While these two diseases share a common symptoms including tremor, it is important to diagnose these two diseases with confidence because successful treatment depends on specific medications. We proposed a novel measurement that can be used for discriminating PD from ET. This measurement quantifies the variation in a timely manner instead of providing a summarized quantity such as root-mean-square of tremor amplitude. Furthermore, an ellipse area covering 95 % of the two-dimensional signal excludes outliers and provides a more reliability of the discrimination. The result shows that the temporal fluctuation while performing resting task can be used to differentiate PD from ET. Furthermore, the differentiation can be improved by applying the fluctuation ratio of the individual subject. As a result, two-sample t test results were reduced while using the fluctuation ratio. The fluctuation ratio acquired from the x-axis of a sensor provides the highest separation of PD to ET tremor compared to other two axes. This is because subjects with tremor have maximum angular movements around this axis compared to the other two. This feature can be applied to classifiers such as neural networks and support vector machines. However, with a simple classification rule, the validation shows that the proposed method can differentiate mild tremor or early stage PD from ET subjects with 100 % accuracy in the 5 subjects in the validation group. While the method would have to be validated with a larger number of subjects, these preliminary results show the feasibility of the approach. This demonstration introduces the efficacy of the proposed method for differential diagnosis of early cases of PD and ET. This method also provides convenience to patients since it takes only 10 s for each task. The total signal collection time including setup and giving instruction is normally less than 10 min. In future work, a larger number of participants are needed to verify whether this feature can represent each group effectively. Furthermore, this novel feature can then be explored with particular tasks designed for more convenient devices such as a smartphone and a smartwatch to obtain tremor data.

## Abbreviations

PD: Parkinson’s disease
ET: essential tremor
UKPDSBB: United Kingdom Parkinson’s Disease Society Brain Bank
DOF: degree of freedom
IMU: inertial measurement unit
TF: temporal fluctuation
FR: fluctuation ratio
TP: true positive
TN: true negative
FP: false positive
FN: false negative
EMG: electromyogram
RMS: root mean square
AR: autoregressive
CSV: comma separated value
TRS: Fahn–Tolosa–Marin Tremor Rating Scale

## References

[1] Lang AE, Lozano AM (1998). Parkinson’s disease. First of two parts. N Engl J Med.

[2] Rajput AH, Rozdilsky B, Rajput A (1991). Accuracy of clinical diagnosis in parkinsonism—a prospective study. Can J Neurol Sci.

[3] Hughes AJ, Daniel SE, Kilford L, Lees AJ (1992). Accuracy of clinical diagnosis of idiopathic Parkinson’s disease: a clinico-pathological study of 100 cases. J Neurol Neurosurg Psychiatry.

[4] Hughes AJ, Daniel SE, Blankson S, Lees AJ (1993). A clinicopathologic study of 100 cases of Parkinson’s disease. Arch Neurol.

[5] Hughes AJ, Daniel SE, Ben-Shlomo Y, Lees AJ (2002). The accuracy of diagnosis of parkinsonian syndromes in a specialist movement disorder service. Brain.

[6] Meara J, Bhowmick BK, Hobson P (1999). Accuracy of diagnosis in patients with presumed Parkinson’s disease. Age Ageing.

[7] Bhidayasiri R (2005). Differential diagnosis of common tremor syndromes. Postgrad Med J.

[8] Wu Y, Ding J, Gao Y, Chen S, Li L, Li R (2013). Mini Review: linkages between essential tremor and Parkinson’s disease?. Front Cell Neurosci.

[9] Hoehn MM (1985). Result of chronic levodopa therapy and its modification by bromocriptine in Parkinson’s disease. Acta Neurol Scand.

[10] Wharrad HJ, Jefferson D (2000). Distinguishing between physiological and essential tremor using discriminant and cluster analyses of parameters derived from the frequency spectrum. Hum Mov Sci.

[11] Poon C, Robichaud JA, Corcos DM, Goldman JG, Vaillancourt DE (2011). Combined measures of movement and force variability distinguish Parkinson’s disease from essential tremor. Clin Neurophysiol.

[12] Ruonala V, Meigal A, Rissanen SM, Airaksinen O, Kankaanpää M, Karjalainen PA (2014). EMG signal morphology and kinematic parameters in essential tremor and Parkinson’s disease patients. J Electromyogr Kinesiol.

[13] Wile DJ, Ranawaya R, Kiss ZH (2014). Smart watch accelerometry for analysis and diagnosis of tremor. J Neurosci Methods.

[14] Engin M, Demirag S, Engin EZ, Celebi G, Ersan F, Asena E, Colakoglu Z (2007). The classification of human tremor signals using artificial neural network. Expert Syst Appl.

[15] Rissanen SM, Kankaanpää M, Meigal A, Tarvainen MP, Nuutinen J, Tarkka IM, Airaksinen O, Karjalainen PA (2008). Surface EMG and acceleration signals in Parkinson’s disease: feature extraction and cluster analysis. Med Biol Eng Comput.

[16] Hossen A, Muthuraman M, Raethjen J, Deuschl G, Heute U (2010). Discrimination of Parkinsonian tremor from essential tremor by implementation of a wavelet-based soft-decision technique on EMG and accelerometer signals. Biomed Signal Process Control.

[17] Woods AM, Nowostawski M, Elizabeth AF, Martin P (2014). Parkinson’s disease and essential tremor classification on mobile device. Pervasive Mob Comput.

[18] Ai L, Wang J, Wang X (2008). Multi-features fusion diagnosis of tremor based on artificial neural network and D–S evidence theory. Sig Process.

[19] Brittain JS, Cagnan H, Mehta AR, Saifee TA, Edwards MJ, Brown P (2015). Distinguishing the central drive to tremor in Parkinson’s disease and essential tremor. J Neurosci.

[20] Bhidayasiri R, Petchrutchatachart S, Pongthornseri R, Anan C, Dumnin S, Thanawattano C (2014). Low-cost, 3-dimension, office-based inertial sensors for automated tremor assessment: technical development and experimental verification. J Parkinsons Dis.

[21] Okada K, Hando S, Teranishi M, Matsumoto Y, Fukumoto I (2001). Analysis of pathological tremors using the autoregression model. Front Med Biol Eng.

[22] Hayes M (1996). Statistical digital signal processing and modeling.

